# Expression pattern of olfactory receptor genes in human cumulus cells as an indicator for competent oocyte selection

**DOI:** 10.3906/biy-2003-79

**Published:** 2020-12-14

**Authors:** Neda DAEI-FARSHBAF, Reza AFLATOONIAN, Fatemeh-Sadat AMJADI, Sara TALEAHMAD, Mahnaz ASHRAFI, Mehrdad BAKHTIYARI

**Affiliations:** 1 Department of Anatomy, Faculty of Medicine, Iran University of Medical Sciences, Tehran Iran; 2 Department of Endocrinology and Female Infertility, Reproductive Biomedicine Research Center, Royan Institute for Reproductive Biomedicine, Academic Center for Education, Culture and Research, Tehran Iran; 3 Cellular and Molecular Research Center, Faculty of Medicine, Iran University of Medical Sciences, Tehran Iran; 4 Department of Molecular Systems Biology, Cell Science Research Center, Royan Institute for Stem Cell Biology and Technology (RI-SCBT), Academic Center for Education, Culture and Research, Tehran Iran; 5 Department of Obstetrics and Gynecology, Faculty of Medicine, Iran University of Medical Sciences, Tehran Iran

**Keywords:** Olfactory receptors, odorant receptors, G-protein coupled receptors, adenylyl cyclase type 3, olfactory marker protein

## Abstract

Odorant or olfactory receptors are mainly localized in the olfactory epithelium for the perception of different odors. Interestingly, many ectopic olfactory receptors with low expression levels have recently been found in nonolfactory tissues to involve in local functions. Therefore, we investigated the probable role of the olfactory signaling pathway in the surrounding microenvironment of oocyte. This study included 22 women in intracytoplasmic sperm injection cycle. The expression of olfactory target molecules in cumulus cells surrounding the growing and mature oocytes was evaluated by Western blotting and real-time polymerase chain reaction. Additionally, integrated bioinformatics analyses were carried out and 6 ectopic olfactory receptors were selected for further evaluation. The initiation of olfactory transduction cascade in cumulus cells of competent oocytes was confirmed by analyzing the expression of adenylyl cyclase type 3 and olfactory market protein. Moreover, the expression pattern of the selected olfactory receptors was evaluated and OR10H2 was selected due to a high level of expression in mature fertile oocytes. We suggested that OR10H2 could be considered as a reliable biomarker for oocyte selection in assisted reproduction technique programs. However, further studies are required to elucidate the role of olfactory transduction cascade in embryo quality and implantation.

## 1. Introduction

Fertilization is a biological process that occurs only after sperm-egg recognition (Kim et al., 1996). The molecular communication between sperm and egg has been extensively studied and sperm chemotaxis is considered as a critical chemical phenomenon (Yoshida and Yoshida, 2011). Despite decades of research, just a few studies showed that the mechanism of chemotactic responses depends on the activation of sperm olfactory receptor coupled to a cAMP-producing machinery to trigger intracellular Ca2+ elevation needed for the sperm movement (Antal et al., 2017; Milardi et al., 2018).

Olfactory or odorant receptors (ORs) are a supergene category of the G-protein-coupled receptors (GPCRs) family with approximately 400 functional genes and about 600 pseudogenes in human genome (Flegel et al., 2013). Upon the binding of odors, the activated OR promotes the stimulation of downstream components including olfactory G-protein (Golf), adenylate cyclase type 3 (AC3), and cyclic nucleotide-gated (CNG) cation channels to induce an elevation in intracellular cAMP amount and finally in intracellular Ca2+ and Na+ concentrations for membrane depolarization (Kang et al., 2015). The accumulation and removal of cAMP and Ca2+ are very critical for accurate activation and termination of the olfactory signaling cascade (Kang et al., 2015; Dibattista and Reisert, 2016). Olfactory marker protein (OMP), a hallmark of olfactory sensory neurons, has the potential of clearing the elevated Ca2+ and acts as a brake on the up-stream of cAMP production to control appropriate concentrations of cAMP following the odor perception (Kang et al., 2015; Dibattista and Reisert, 2016). Therefore, it is generally accepted that OMP is indeed an important component in modulating olfactory transduction (Albeanu et al., 2018).

Odorant receptors are routinely localized in sensory organs like the olfactory epithelium for perception of different odors and pheromones (Ferrer et al., 2016). Interestingly, many ORs have recently been found in nonolfactory tissues with negligible expression levels to detect extracellular cues and are involved in local physiological functions other than olfaction (Kang et al., 2015; Ferrer et al., 2016). The physiological functions of ectopic ORs in human reproductive system are largely unknown. However, the results of recent studies on sperm have identified that the significant role of these ORs is chemotaxis toward follicular fluid-derived chemo-attractants (Teves et al., 2009; Yoshida and Yoshida, 2011; Hunter and Gadea, 2014). The chemotactic response of spermatozoa is regulated by OR-mediated intracellular Ca2+ elevation needed for asymmetric flagellar beating (Yoshida and Yoshida, 2011). Ca2+ current is highly important for not only chemotaxis but also many crucial events of reproduction like oocyte maturation, fertilization, and also embryo development (Carvacho et al., 2018). In addition to Ca2+ regulation, the activated ORs could participate in cytokinesis by exerting a fundamental role on cytoskeleton remodeling, just like the phenomenon that oocytes need to mature (Milardi et al., 2018; Zhang et al., 2019).

Taken together, we hypothesized that the olfactory signaling cascade could be initiated with the binding of ligands existent in the surrounding microenvironment to probable ORs on oocyte to promote maturation through cytoskeleton remodeling and ion regulation. In view of the bidirectional cumulus-oocyte relationship and the need for noninvasive prognostic markers for competent oocyte selection, the present study aims to verify whether the expression level of some olfactory receptor genes in cumulus cells (CCs) can be a predictor of oocyte quality under assisted reproductive technique (ART) programs (Hosseini et al., 2016; Salehi et al., 2017). Since OR-mediated events are modulated by well-conserved downstream components, we simultaneously assessed the expression pattern of 6 ectopically expressed ORs and downstream genes in human cumulus cells using real-time quantitative polymerase chain reaction (qPCR). Furthermore, in order to overcome the limitations of using OR as a target protein, the current study used Western blotting to detect AC3 and OMP proteins, which are potential targets to screen for OSC in nonolfactory tissues (Kang et al., 2015; Albeanu et al., 2018).

## 2. Method and materials

### 2.1. Patient preparation

The study included 22 healthy women donors in intracytoplasmic sperm injection (ICSI) cycles to collect CCs at Shahid Akbarabadi Hospital (Tehran, Iran) after signing informed consent. This study was approved by the Ethics Committees of Iran University of Medical Sciences (IUMS), Tehran, Iran (approval date 25/07/2016, IUMS. 1395.9221113202). All the samples were obtained from the patients after signing the informed consent. Women with the history of Cushing’s syndrome, diabetes mellitus, congenital adrenal hyperplasia, polycystic ovary syndrome, and endometriosis, and those aged over 36 years were excluded from the study. Controlled ovarian hyperstimulation was performed using a gonadotrophin-releasing hormone agonist (Buserelin Acetate Suprefact, Aventis, Germany) as the long protocol.

### 2.2. Semen analysis

Severe male factor infertility was defined according to the WHO criteria, the Kruger’s criteria (Menkveld, 2010), and the TUNEL test (Sharma et al., 2010). DNA fragmentation analysis was carried out using the In Situ Cell Death Detection Kit (Roche, Mannheim, Germany) according to the manufacturer’s instructions. The prepared samples were examined using the flow cytometry technique and TUNEL-positive sperms in each population were measured in the corresponding histogram. The negative and positive controls were prepared by omitting terminal deoxy transferase (TdT) enzyme and incubating with DNase I solution (Sigma, Germany), respectively.

### 2.3. Collection of cumulus cells

**Figure 1:**
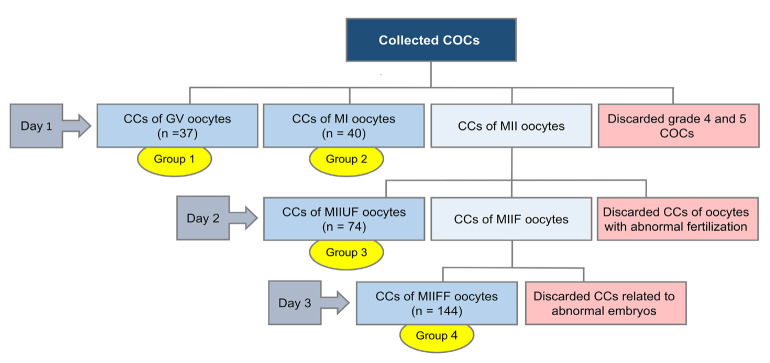
The classification of cumulus cells. Day 1: Oocyte quality analysis and ICSI, Day2: ICSI outcome and fertilization assessment, Day 3: Embryo quality and cleavage assessment. COCs: Cumulus- Oocyte Complexes, CCs: Cumulus Cells, GV: Germinal vesicle, MI: Metaphase I, MII: Metaphase II,MIIF: Metaphase II Fertilized, MIIUF: Metaphase II Unfertilized, MIIF: Metaphase II Fertilized, MIIFF: Metaphase II Final Fertilized.

Following follicular puncture, each cumulus-oocyte complex (COC) was scored from 1 to 5 according to the morphological characteristics of the ooplasm, the mass of CCs, corona radiata, and detached membrana granulosa cells based on the grading system of Lin et al. (Salehi et al., 2017). The CCs of grade 4 and 5 COCs were excluded from the study. Denudation was performed mechanically, and the CCs associated with each oocyte were aspirated from drops. The collected cells were centrifuged twice with PBS at 800 g for 8 min, labeled, and stored individually at −80 °C until further assessment. The classification of CCs was performed based upon the nuclear maturity (Ozgur et al., 2015), the fertilization quality (Hosseini et al., 2016), and also the cleavage status (Magli et al., 2012) (Figure 1). Briefly, at the time of ICSI (day 1), the oocytes were graded according to the nuclear status into 3 groups: (i) immature oocyte at the GV stage (nGV = 37); (ii) immature oocyte (MI) without the first polar body or GV (nMI = 40); and (iii) mature oocyte in MII with the first polar body (MII). On day 2, the fertilization potential (2 pronuclei (2PN) formation) was evaluated and the CCs of oocytes without 2PN were classified as the unfertilized MII group (nMIIUF = 74). The oocytes with normal fertilization were cultured individually and the CCs associated with oocytes with abnormal fertilization and micronuclei were discarded. On day 3, the cleavage quality of the individually cultured embryos was examined in terms of fragmentation rate, the number of normal blastomeres, and the presence of multinucleated blastomeres. The CCs associated with embryos with <20% fragmentation, no multinucleated blastomeres, and 4 regular blastomeres were selected as the final fertilized MII group (nMIIFF = 144).

### 2.4. RNA isolation and reverse transcription PCR

The extraction process was performed by Trizol reagent (Sigma Pool, UK) to extract the RNA and proteins of an individual sample simultaneously. To isolate the total RNA, the collected cumulus cells were lysed in Trizol reagent and centrifuged after adding chloroform. Out of the 3 phases that appeared (Trizol-chloroform fractions), the upper aqueous phase was processed and the extracted total RNA was treated using DNase (Fermentas, Sankt Leon-Rot, Germany) and evaluated using the NanoDrop spectrophotometer (Thermo Scientific, NanoDrop 2000 spectrophotometer). After that, the first-strand complementary DNA (cDNA) was synthesized to perform a reverse transcription PCR (RT-PCR) with human-specific primers. PCR products were assessed using gel electrophoresis to verify the characteristics of forward and reverse primers (Table S1). GAPDH was used as a housekeeping control and all the experiments included RT and negative controls.

### 2.5. Real-time PCR

Real-time quantitative PCR reactions were carried out by an ABI Prism 7300Sequence Detector (Applied Biosystems, Foster City, CA, USA) for mRNA quantification and were repeated three times. Thermal cycling conditions for 45 cycles were denaturation at 95 °C for 30 s, annealing at 60 °C for 30 s, and extension at 72 °C for 30 s. To validate primer specificities, melting curve analyses were carried out. Standard curves were obtained for each gene to verify primer efficiency using the logarithmic dilution series of the total cDNA [25]. The expression level of OMP and AC3 and the selected OR genes were determined according to the algorithm of 2-∆∆CT. GraphPad Prism 8 software 8.0.1 was used to draw heat-map for analyzing expression pattern.

### 2.6. Protein extraction and western blotting

The protein of CCs was extracted using Trizol reagent (Sigma Pool, UK) in order to perform a Western blotting analysis. Briefly, 100% ethanol was added to the tube containing the organic phase and a small interphase (see section 2.4: Trizol-chloroform fractions) to precipitate DNA. The tubes were centrifuged at 2000 g for 5 min at 4 °C and supernatant was used for protein extraction. The protein was precipitated using isopropanol and 10 min of incubation at room temperature. In the next step, the tubes were centrifuged at 12,000 g for 10 min and the pellets were washed 3 times using 0.3 M guanidine hydrochloride in 95% ethanol. In each wash, the samples were incubated for 20 min at room temperature and centrifuged at 7500 g for 5 min at 4 °C. Subsequently, 100% ethanol was added to the pellets, followed by an incubation for 20 min at room temperature and centrifuged at 7500 g for 5 min at 4 °C. The supernatant was removed and the 2% SDS lysing buffer was added to the protein pellets. The extracted protein levels were determined using the Bradford Assay (Bio-Rad Protein Assay Dye Reagent Concentrate; Bio-Rad Laboratories, Inc.) according to the manufacturer’s instructions. Then, 5 μL of protein mixture was separated from each sample by sodium dodecyl sulfate-polyacrylamide electrophoresis (SDS-PAGE) on 12.5% polyacrylamide gel and transferred onto polyvinylidene difluoride (PVDF) membrane (Bio-Rad, USA). The membrane was blocked by 5–10% (w/v) nonfat dried milk and incubated with primary antibody against OMP (Santa Cruz Biotechnology, Santa Cruz, CA), AC3 (Abcam, Cambridge, Cambridgeshire, UK), GAPDH (Abcam), and the secondary antibody. The developing process was performed and the peroxidase activity was visualized to provide a permanent record. The films were scanned by a densitometer (GS-800, BioRad) and the protein expression levels were analyzed using Image J software.

### 2.7. Olfactory receptor selection

**Figure 2:**
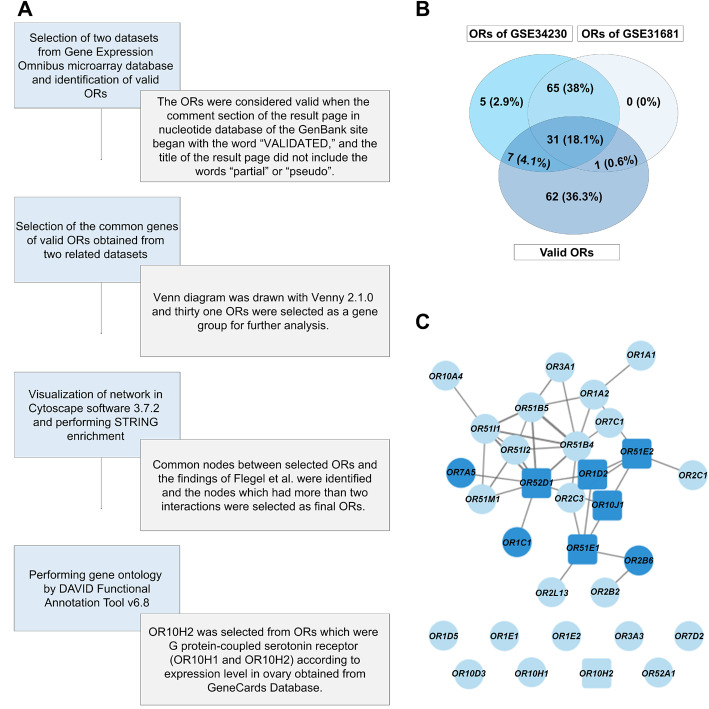
Olfactory receptor selection. (A) The Flowchart of olfactory receptor (OR) selection process in detail. (B) Venn diagram of selected ORs; Overlapping thirty-one genes, as the common and valid ORs, were taken into account for further analysis. (C) The network of thirty-one ORs drawn with Cytoscape software. Nods represent genes; edges represent interactions. Dark colored nodes are olfactory receptors with a special function in human sperm. All rectangle nodes are the final selected ORs.

The gene expression profiles were searched from the Gene Expression Omnibus microarray database (GEO) using the keywords “cumulus cells” and “Homo sapiens” (Figure 2A). GSE34230 and GSE31681 datasets were selected and valid ORs (Table S2) were identified according to the nucleotide database of the GenBank site (Kang et al., 2015). Then, common genes among valid olfactory receptors of 2 data series (Table S3) were identified using a Venn diagram drawn with Venny 2.1.0 (Figure 2B). For further analyses, 31 ORs were selected as a gene group by Cytoscape software 3.7.2 (Cline et al., 2007) and DAVID Functional Annotation Tool v6.8. We visualized the Cytoscape network and selected 5 nodes in common with ORs, which play a role in human sperm physiology (Figure 2C) (Flegel et al., 2016). In addition, OR10H2 was selected after the analysis of the DAVID gene due to its functioning as a G protein-coupled serotonin receptor (Figure S1). Therefore, the OR10H2 analysis could reveal the probable role of serotonin in oocyte maturation as one of the main modulators of female sexual behavior (Uphouse, 2014).

### 2.8. Statistics

The statistical analyses were performed using the IBM SPSS software (version 24). To make comparisons between the groups, independent t-test or Mann–Whitney U test were used. Analysis of variance (ANOVA test) was performed to compare quantitative variables between the groups. All the experiments and analyses were performed 3 times and P-values ≤ 0.05 were considered to be statistically significant.

## 3. Results

### 3.1. Demographic data

The hormonal and demographic characteristics of the women taking part in this study were in the normal range, which are presented in Table 1.

**Table 1:** The comparisons related to demographic and hormonal data.

Characteristics	Mean ± SD
Age (years)	31.83 ± 3.32
BMI (kg/m2)	23.38 ± 2.13
FSH (mIU/mL)	5.82 ± 1.36
LH (mIU/mL)	4.59 ± 1.53
TSH (mlU/L)	1.77 ± 0.48
PRO (µg/L)	16.07 ± 7.65
AMH (ng/mL)	2.42 ± 0.60
Number of oocytes	14.13 ± 2.52
Data are expressed as mean ± SD.BMI: Body mass index, FSH: Follicle-stimulating hormone, LH: Luteinizing hormone, TSH: Thyroid stimulating hormone, PRL: Prolactin, AMH: Anti-Müllerian hormone.	

### 3.2. Spermogram and TUNEL data

**Figure 3:**
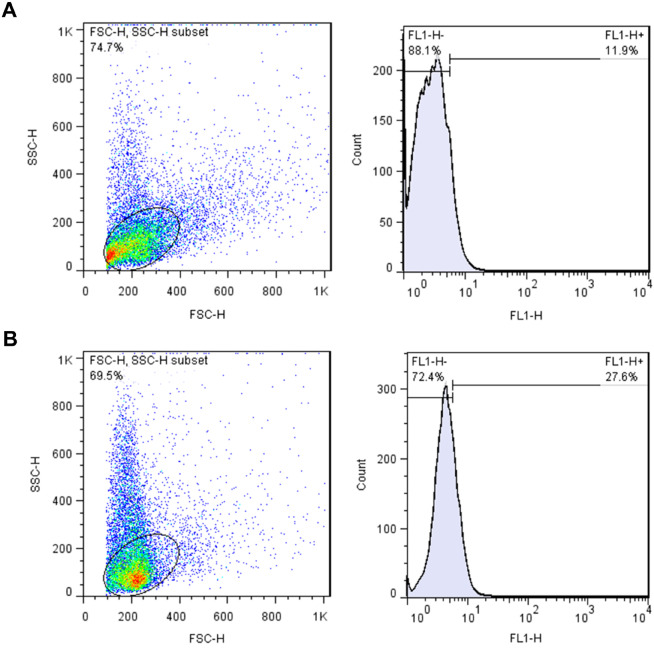
TUNEL assay. (A) example of a sample with low level (11.9%) of TUNEL-positive sperms; (B) example of a sample with high proportion (27.6%) of TUNEL-positive sperms. FSC: Forward-angle light scatter, SSC: Side-angle light scatter, FL1-H: Fluorescence intensity.

Table 2 presents the results of the spermogram and TUNEL test. The sperm DNA fragmentation index (DFI) was calculated according to the TUNEL test cutoff point, which was considered to be less than 19.25% (Figure 3A) and the samples with a high level of TUNEL positivity were excluded from the study (Figure 3B).

**Table 2:** Semen characteristics and DFI evaluation.

Parameters	Vol (mL)	Conc. (106/mL)	T motility (%)	P motility (%)	Morph (%)	DFI (%)
Mean ± SD	3.63 ± 1.48	73.68 ± 22.61	62.9 ± 8.8	45 ± 7.5	5.6 ± 1	9.68 ± 3.18
At least two independent replicates were processed separately and analyzed for each semen sample.DFI: DNA Fragmentation Index, Vol: Volume, Conc.: Concentration, T motility: Total motility, P motility: Progressive motility, Morph: Morphology.						

### 3.3. Gene expression levels for AC3 and OMP

**Figure 4:**
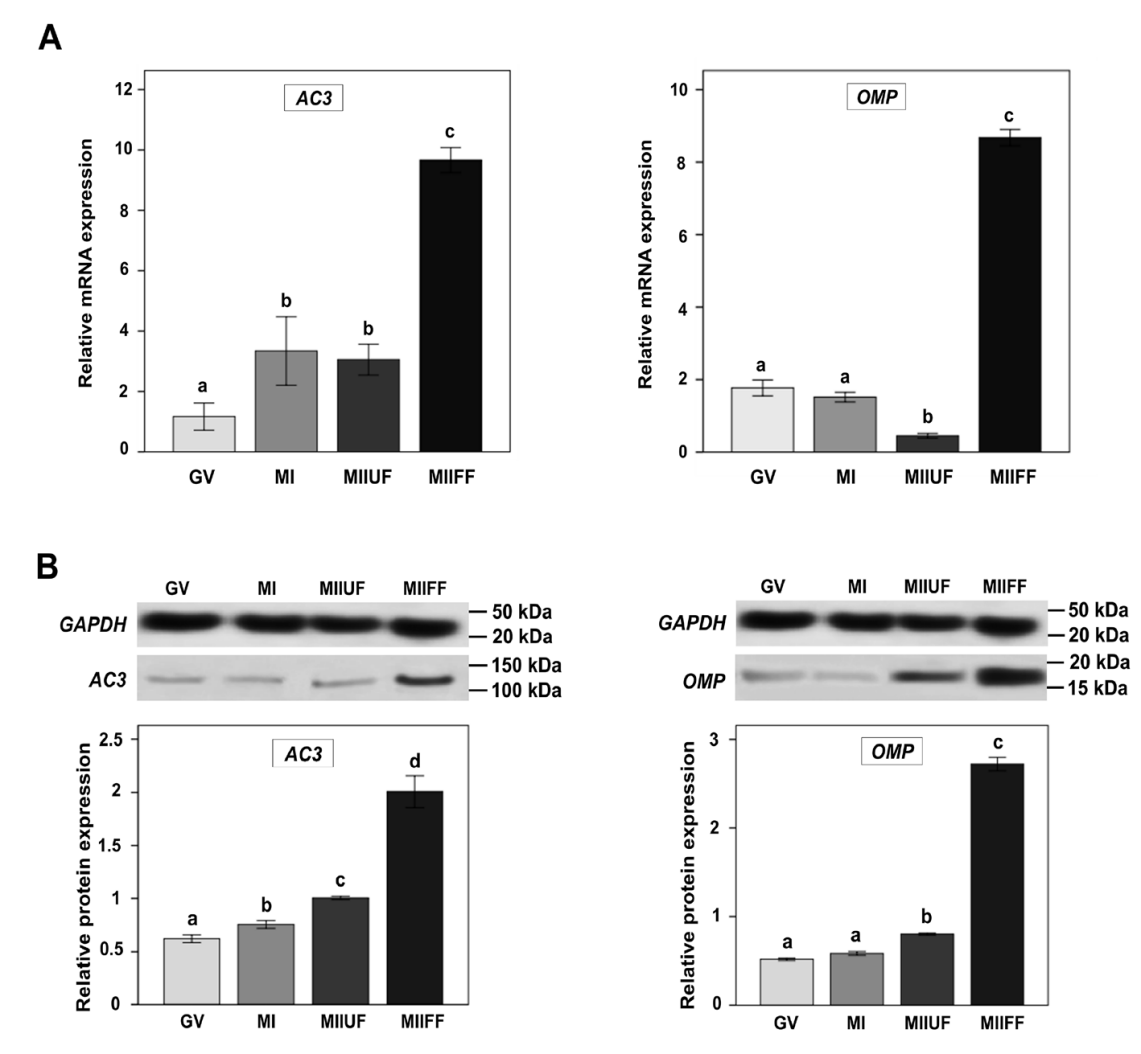
Relative mRNA and protein expression of AC3 and OMP in human cumulus cells. (A) The relative mRNA levels (mean ± SD) represent expression amount of AC3 and OMP genes, which were normalized to GAPDH. The minimum number of biological replicates for each gene was 10 samples per group, with three technical replicates. (B) The expression of proteins was confirmed by western blot in cumulus cells of GV, MI, MIIUF, and MIIFF oocytes. Protein ratios of AC3 and OMP were normalized to GAPDH. Three biological replicates were run in triplicates and values are expressed as means ± SD. The statistical difference between two different lowercase letters such as “a’’ and “b’’, “a’’ and “c’’, or “b’’ and “c’’ represents P-value ≤ 0.05. AC3: Adenylyl Cyclase type 3, OMP: Olfactory marker protein, GV: Germinal vesicle, MI: Metaphase I, MII: Metaphase II, MIIUF: Metaphase II Unfertilized, MIIF: Metaphase II Fertilized, MIIFF: Metaphase II Final Fertilized.

All the amplified RT-PCR products were at the expected size (Table S1) for GAPDH, AC3, OMP, and the selected ORs. To assess the activation of the olfactory signaling cascade in CCs during oogenesis, we performed a qPCR analysis for AC3 and OMP genes as target downstream genes in the OR-mediated pathways. As shown in Figure 4A, higher expression levels for the AC3 and OMP genes were found in the CCs of the final fertilized MII oocytes (MIIFF) compared to others.

### 3.4. Protein expression of AC3 and OMP

To confirm the results of the qPCR analyses, the AC3 and OMP proteins were analyzed by Western blotting in the CCs of different maturation stages (Figure 4B). The expression in the final fertilized MII groups was significantly higher than the other 3 stages.

### 3.5. Expression pattern of selected ORs

**Figure 5:**
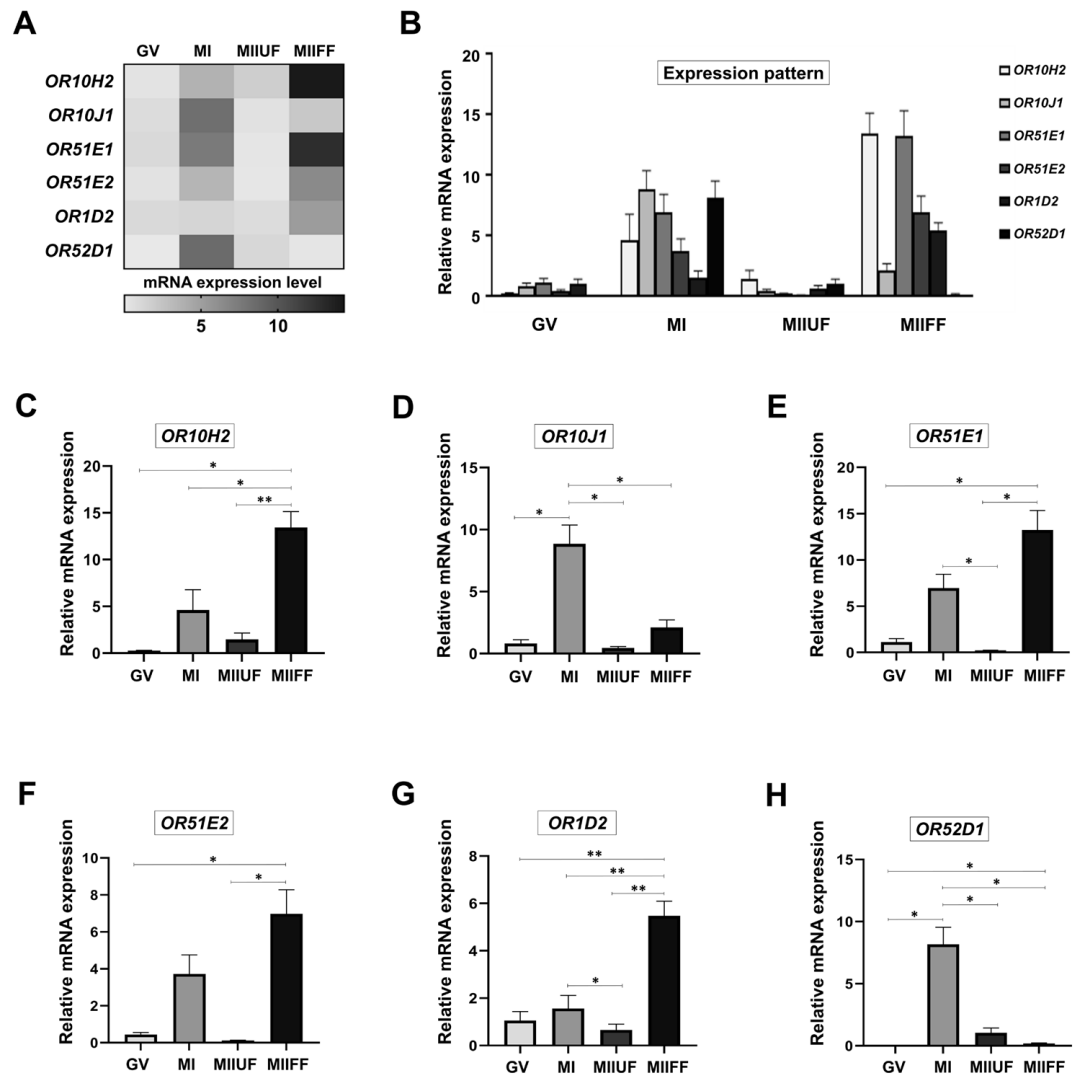
The patterned expression of selected olfactory receptor genes in human cumulus cells. (A) Heat-map and (B) Bar graph show the patterned expression of selected ORs in cumulus cells from oocytes in different stages of meiotic maturation. The expression level of (C) OR10H2, (D) OR10J1, (E) OR51E1, (F) OR51E2, (G) OR1D2, and (H) OR52D1 after real-time PCR analysis. The relative mRNA levels represent the amount of mRNA expression normalized to GADPH, after which the data were used to calculate relative expression levels. The experiment was conducted in triplicate, and the data are expressed as mean ± SD in bar graphs and as mean in heat-map. P-values less than 0.05 and 0.01 are given one (*) and two (**) asterisks, respectively. All experiments were repeated thrice for at least 10 biological replicates per group. GV: Germinal vesicle, MI: Metaphase I, MIIUF: Metaphase II Unfertilized, MIIF: Metaphase II Fertilized, MIIFF: Metaphase II Final Fertilized.

Detailed analysis of ORs mRNA expression level in CCs demonstrated an exclusive patterned arrangement in different stages of oocyte development (Figures 5A and 5B). Detailed evaluation of statistical difference and relative mRNA expression level revealed that OR10H2 might be indicative of high fertilization potential in related MII oocyte (Figures 5C–5H).

## 4. Discussion

The current study demonstrated the fundamental role of the olfactory transduction pathway during oogenesis in oocyte maturation and the acquisition of fertilization potential. More importantly, the provided expression pattern of some ectopically expressed ORs revealed OR10H2 as a biomarker for high quality and fertilizability in the associated MII oocyte.

Olfactory receptors, which belong to the largest category of human chemoreceptors, could be expressed ectopically in nonolfactory tissues for specific physiological functions (Flegel et al., 2016). To accomplish a local function, ectopic ORs employ several mechanisms for calcium ion influx, including AC3 activation to increase cAMP concentration and/or direct activation of calcium channels, specially L-type channels, using inositol-1, 4, 5-trisphosphate (IP3) receptor, and phospholipase C (Horner et al., 2003; Flegel et al., 2016). Therefore, the present study employed AC3 and OMP among several components of the olfactory signaling pathway due to their direct effects in cAMP and calcium regulation (Sen and Caiazza, 2013; Kang et al., 2015; Dibattista and Reisert, 2016). The selection of AC3 and OMP to confirm the olfactory pathway function could overcome the limitations related to the availability of specific antibodies for ORs due to their structural similarity (Kang and Koo, 2012).

The expression level of AC3 and OMP in the cumulus cells (Figure 4A) convinced us about the role of the olfactory signaling pathway in oocyte maturation and quality acquisition. We found an elevated expression of the AC3 and OMP genes in the MIIFF group, which could reveal that AC3 activation is an OR-dependent manner and its level is controlled by OMP in the cumulus cells of fertile MII oocytes. According to our results, it seems that other mechanisms, regulators, and maybe other types of adenylyl cyclase are responsible for maturation events in the GV stage. These results, which were consistent with the findings of Sen and Caiazza (Sen and Caiazza, 2013), were also confirmed by our Western blotting analysis (Figure 4B). Consequently, it is concluded that the olfactory pathway is mainly activated in the cumulus cells of competent MII oocytes and plays critical roles in the maturation process along with AC3 and OMP.

The main aspects of the present study were to assess the expression level of 6 OR genes and to illustrate their specific expression patterns in a different stage of oocyte maturation. Although the ectopic expression and the exclusive pattern of ORs in human cumulus cells were confirmed in this study (Figure 5), how disorders related to infertility alter the expression pattern of ORs and how ORs are regulated by gonadotropins or steroids remain to be determined. Additionally, the patterns that were drawn showed that the expression of the selected ORs was dominant from the MI stage onward, which confirmed the involvement of other GPCRs in the phenomena of the GV stage.

Based on our detailed analysis, OR10H2 could be considered as a reliable biomarker gene in fertile oocyte selection during ARTs (Figures 5). OR10H2 as a G protein-coupled serotonin receptor (Figures SI) could bind to the secreted serotonin and maintain the oocyte development and fertilization potential through the activation of AC3 and cAMP production in cumulus cells (Doty and Kamath, 2014; Soonthornsumrith et al., 2018). Giacomo et al. have shown that cAMP elevation prevents cumulus cell apoptosis and allows them to preserve oocyte fertilizability through exerting protective effects on oocyte (Di Giacomo et al., 2016). Taken together, it is concluded that the activation of OR10H2 through serotonin promotes successful fertilization by preventing cumulus cell senescence and improving oocyte survival.

Furthermore, it is likely that the serotonin-mediated OR10H2 stimulation and the subsequent OMP activation in competent oocytes trigger an OR-dependent signal transduction cascade between the nervous and endocrine systems to help ovarian steroid secretion and to enhance oocyte quality (Kang et al., 2015; Soonthornsumrith et al., 2018). Given the previous findings, serotonin could also regulate progesterone secretion by granulosa cells (Yang et al., 2015). Since cumulus cells synthesize and secrete progesterone continuously as a sperm chemoattractant factor after ovulation (Guidobaldi et al., 2008; Sun and Diaz, 2019), it seems that the cumulus cells of competent MII oocytes could secrete an acceptable amount of progesterone across the effects of local serotonin, and are more likely to attract sperms especially during natural cycles and maybe in in vitro fertilization (IVF).

Central serotonin regulates sexual behaviors and gonadotropin secretion (Kim et al., 2006; Bhattarai et al., 2014; Uphouse, 2014), but peripheral serotonin in female genital tract, reached through the blood vessels and nerve endings or produced by the surrounding cells, could cause vasodilatory functions and steroidogenesis independently from central serotonin (Frohlich and Meston, 2000; Amireault and Dubé, 2005). Thus, the elevation of local serotonin in ovaries, oviducts, uterus, and even oocytes might improve oocyte maturation and also fertilization potential by the activation of OR10H2. However, further investigations are required on the effect of OR10H2 and its interaction with serotonin on oocyte quality.

## 5. Conclusion

To the best of our knowledge, this was the first attempt to study the function of the olfactory transduction pathway in oocyte maturation and fertilization outcome. Based on our results, we suggest that OR10H2 could be considered as a reliable biomarker in oocyte selection during ART programs. Moreover, it is possible that activation of OR10H2 by peripheral serotonin might help ovarian steroidogenesis, enhance oocyte maturation, and improve fertilization competence.

Supplementary Materials
