# Supplementary material for: Expression pattern of olfactory receptor genes in human cumulus cells as an indicator for competent oocyte selection
Source: Turk J Biol. 2020 Dec 14;44(6):371–80. doi: 10.3906/biy-2003-79 (PMC7759194; doi:10.3906/biy-2003-79)
Supplement: Supplementary file 1 — Supplementary Materials [file turkjbio-44-371-sup001.pdf]

Supplementary data

Table S1. Characteristics of the primers used for qPCR.

**Gene**	**Forward primer (5'–3')**	**Reverse primer (5'–3')**	**Product size (bp)**	**Annealing temperature (°C)**
GAPDH	CTCATTTCCTGGTATGACAACGA	CTTCCTCTTGTGCTCTTGCT	122	60
AC3	GGAACGTGTCTATGAGATGCGA	TAGAAGTCAGCAAAGTTGGGCA	174	60
OMP	AGGACTCGGATGCCATAGATTG	GGAAGTACATGACCTTGCGGAT	96	60
OR10J1	CCCTGTGGTATACACCCTGAGA	TGCCTAGTGTGGATGTTGACAG	123	60
OR10H2	ACCTTCCTCAGCACACTCTATTC	GGTGATGGTCATCCTCCTATCAT	87	60
OR1D2	TACTCCTTTCCTTGTGTTGGGT	TGCCATCCTCAGCAATACATACAT	137	60
OR51E1	CTTATCGTCATCATCTCCGCCA	TGTCAAGCCCAACACAGTCTTA	90	60
OR51E2	TGTGGTACTCGCCTTCTATGTG	CACCCGTGTTCTGATCTGTTTG	172	60
OR52D1	TCCTGGTTTTCTACATCCCTGC	TCCGAATCTCCTTGGTTCTAGC	162	60

Table S2. Valid olfactory receptor genes according to NCBI. The following three steps were performed for all human ORs to determine the valid ones and limit the number of selected ORs for further evaluations: (1) The database of the GenBanksite (http://www.ncbi.nlm.nih.gov/genbank) was searched for the name of relate olfactory genes (2) In the search result, the mRNA sequence, was selected. (3) The OR was considered valid when the comment section of the result page began with the word "VALIDATED,".

**OR10A4:** Olfactory Receptor family 10 subfamily A member 4	**OR4N2:** Olfactory Receptor family 4 subfamily N member 2
**OR10D3:** Olfactory Receptor family 10 subfamily D member 3	**OR4N4:** Olfactory Receptor family 4 subfamily N member 4
**OR10G2:** Olfactory Receptor family 10 subfamily G member 2	**OR4P4:** Olfactory Receptor family 4 subfamily P member 4
**OR10G3:** Olfactory Receptor family 10 subfamily G member 3	**OR4S2:** Olfactory Receptor family 4 subfamily S member 2
**OR10H1:** Olfactory Receptor family 10 subfamily H member 1	**OR51B4:** Olfactory Receptor family 51 subfamily B member 4
**OR10H2:** Olfactory Receptor family 10 subfamily H member 2	**OR51B5:** Olfactory Receptor family 51 subfamily B member 5
**OR10H4:** Olfactory Receptor family 10 subfamily H member 4	**OR51D1:** Olfactory Receptor family 51 subfamily D member 1
**OR10J1:** Olfactory Receptor family 10 subfamily J member 1	**OR51E1:** Olfactory Receptor family 51 subfamily E member 1
**OR10Q1:** Olfactory Receptor family 10 subfamily Q member 1	**OR51E2:** Olfactory Receptor family 51 subfamily E member 2
**OR10W1:** Olfactory Receptor family 10 subfamily W member 1	**OR51H1:** Olfactory Receptor family 51 subfamily H member 1
**OR11A1:** Olfactory Receptor family 11 subfamily A member 1	**OR51I1:** Olfactory Receptor family 51 subfamily I member 1
**OR13A1:** Olfactory Receptor family 13 subfamily A member 1	**OR51I2:** Olfactory Receptor family 51 subfamily I member 2
**OR13H1:** Olfactory Receptor family 13 subfamily H member 1	**OR51M1:** Olfactory Receptor family 51 subfamily M member 1
**OR14A2:** Olfactory Receptor family 14 subfamily A member 2	**OR51V1:** Olfactory Receptor family 51 subfamily V member 1
**OR14K1:** Olfactory Receptor family 14 subfamily K member 1	**OR52A1:** Olfactory Receptor family 52 subfamily A member 1
**OR1A1:** Olfactory Receptor family 1 subfamily A member 1	**OR52A5:** Olfactory Receptor family 52 subfamily A member 5
**OR1A2:** Olfactory Receptor family 1 subfamily A member 2	**OR52B6:** Olfactory Receptor family 52 subfamily B member 6
**OR1C1:** Olfactory Receptor family 1 subfamily C member 1	**OR52D1:** Olfactory Receptor family 52 subfamily D member 1
**OR1D2:** Olfactory Receptor family 1 subfamily D member 2	**OR52E2:** Olfactory Receptor family 52 subfamily E member 2
**OR1D5:** Olfactory Receptor family 1 subfamily D member 5	**OR52E5:** Olfactory Receptor family 52 subfamily E member 5
**OR1E1:** Olfactory Receptor family 1 subfamily E member 1	**OR52I1:** Olfactory Receptor family 52 subfamily I member 1
**OR1E2:** Olfactory Receptor family 1 subfamily E member 2	**OR52I2:** Olfactory Receptor family 52 subfamily I member 2
**OR1L1:** Olfactory Receptor family 1 subfamily L member 1	**OR52J3:** Olfactory Receptor family 52 subfamily J member 3
**OR1L6:** Olfactory Receptor family 1 subfamily L member 6	**OR52K1:** Olfactory Receptor family 52 subfamily K member 1
**OR2A2:** Olfactory Receptor family 2 subfamily A member 2	**OR52K2:** Olfactory Receptor family 52 subfamily K member 2
**OR2A4:** Olfactory Receptor family 2 subfamily A member 4	**OR52L1:** Olfactory Receptor family 52 subfamily L member 1
**OR2AP1:** Olfactory Receptor family 2 subfamily AP member 1	**OR52M1:** Olfactory Receptor family 52 subfamily M member 1
**OR2B2:** Olfactory Receptor family 2 subfamily B member 2	**OR52N5:** Olfactory Receptor family 52 subfamily N member 5
**OR2B3:** Olfactory Receptor family 2 subfamily B member 3	**OR56A1:** Olfactory Receptor family 56 subfamily A member 1
**OR2B6:** Olfactory Receptor family 2 subfamily B member 6	**OR56A5:** Olfactory Receptor family 56 subfamily A member 5
**OR2C1:** Olfactory Receptor family 2 subfamily C member 1	**OR56B1:** Olfactory Receptor family 56 subfamily B member 1
**OR2C3:** Olfactory Receptor family 2 subfamily C member 3	**OR5B12:** Olfactory Receptor family 5 subfamily B member 12
**OR2G6:** Olfactory Receptor family 2 subfamily G member 6	**OR5B17:** Olfactory Receptor family 5 subfamily B member 17
**OR2H1:** Olfactory Receptor family 2 subfamily H member 1	**OR5B2:** Olfactory Receptor family 5 subfamily B member 2
**OR2J2:** Olfactory Receptor family 2 subfamily J member 2	**OR5B3:** Olfactory Receptor family 5 subfamily B member 3
**OR2L13:** Olfactory Receptor family 2 subfamily L member 13	**OR5M3:** Olfactory Receptor family 5 subfamily M member 3
**OR2L5:** Olfactory Receptor family 2 subfamily L member 5	**OR5V1:** Olfactory Receptor family 5 subfamily V member 1
**OR2T8:** Olfactory Receptor family 2 subfamily T member 8	**OR6C68:** Olfactory Receptor family 6 subfamily C member 68
**OR2V1:** Olfactory Receptor family 2 subfamily V member 1	**OR6K3:** Olfactory Receptor family 6 subfamily K member 3
**OR2W1:** Olfactory Receptor family 2 subfamily W member 1	**OR6P1**: Olfactory Receptor family 6 subfamily P member 1
**OR3A1:** Olfactory Receptor family 3 subfamily A member 1	**OR7A5**: Olfactory Receptor family 7 subfamily A member 5
**OR3A2:** Olfactory Receptor family 3 subfamily A member 2	**OR7C1**: Olfactory Receptor family 7 subfamily C member 1
**OR3A3:** Olfactory Receptor family 3 subfamily A member 3	**OR7D2**: Olfactory Receptor family 7 subfamily D member 2
**OR4A47:** Olfactory Receptor family 4 subfamily A member 47	**OR8J1:** Olfactory Receptor family 8 subfamily J member 1
**OR4A5:** Olfactory Receptor family 4 subfamily A member 5	**OR8K5:** Olfactory Receptor family 8 subfamily K member 5
**OR4C11:** Olfactory Receptor family 4 subfamily C member 11	**OR8S1:** Olfactory Receptor family 8 subfamily S member 1
**OR4C13:** Olfactory Receptor family 4 subfamily C member 13	**OR8U8:** Olfactory Receptor family 8 subfamily U member 8
**OR4E2:** Olfactory Receptor family 4 subfamily E member 2	**OR9G9:** Olfactory Receptor family 9 subfamily G member 9
**OR4F29:** Olfactory Receptor family 4 subfamily F member 29	**OR9Q1:** Olfactory Receptor family 9 subfamily Q member 1
**OR4K1:** Olfactory Receptor family 4 subfamily K member 1	**OR9Q2:** Olfactory Receptor family 9 subfamily Q member 2
**OR4M2**: Olfactory Receptor family 4 subfamily M member 2	

Table S3. Selected olfactory receptors: common and valid ORs. Among the valid ORs expressed in human cumulus cells, only the commonly expressed ORs were selected from GEO datasets to limit the number of genes for further analyses.

**OR10A4**	Olfactory Receptor family 10 subfamily A member 4
**OR10D3**	Olfactory Receptor family 10 subfamily D member 3
**OR10H1**	Olfactory Receptor family 10 subfamily H member 1
**OR10H2**	Olfactory Receptor family 10 subfamily H member 2
**OR10J1**	Olfactory Receptor family 10 subfamily J member 1
**OR1A1**	Olfactory Receptor family 1 subfamily A member 1
**OR1A2**	Olfactory Receptor family 1 subfamily A member 2
**OR1C1**	Olfactory Receptor family 1 subfamily C member 1
**OR1D2**	Olfactory Receptor family 1 subfamily D member 2
**OR1D5**	Olfactory Receptor family 1 subfamily D member 5
**OR1E1**	Olfactory Receptor family 1 subfamily E member 1
**OR1E2**	Olfactory Receptor family 1 subfamily E member 2
**OR2B2**	Olfactory Receptor family 2 subfamily B member 2
**OR2B6**	Olfactory Receptor family 2 subfamily B member 6
**OR2C1**	Olfactory Receptor family 2 subfamily C member 1
**OR2C3**	Olfactory Receptor family 2 subfamily C member 3
**OR2L13**	Olfactory Receptor family 2 subfamily L member 13
**OR3A1**	Olfactory Receptor family 3 subfamily A member 1
**OR3A3**	Olfactory Receptor family 3 subfamily A member 3
**OR51B4**	Olfactory Receptor family 51 subfamily B member 4
**OR51B5**	Olfactory Receptor family 51 subfamily B member 5
**OR51E1**	Olfactory Receptor family 51 subfamily E member 1
**OR51E2**	Olfactory Receptor family 51 subfamily E member 2
**OR51I1**	Olfactory Receptor family 51 subfamily I member 1
**OR51I2**	Olfactory Receptor family 51 subfamily I member 2
**OR51M1**	Olfactory Receptor family 51 subfamily M member 1
**OR52A1**	Olfactory Receptor family 52 subfamily A member 1
**OR52D1**	Olfactory Receptor family 52 subfamily D member 1
**OR7A5**	Olfactory Receptor family 7 subfamily A member 5
**OR7C1**	Olfactory Receptor family 7 subfamily C member 1
**OR7D2**	Olfactory Receptor family 7 subfamily D member 2

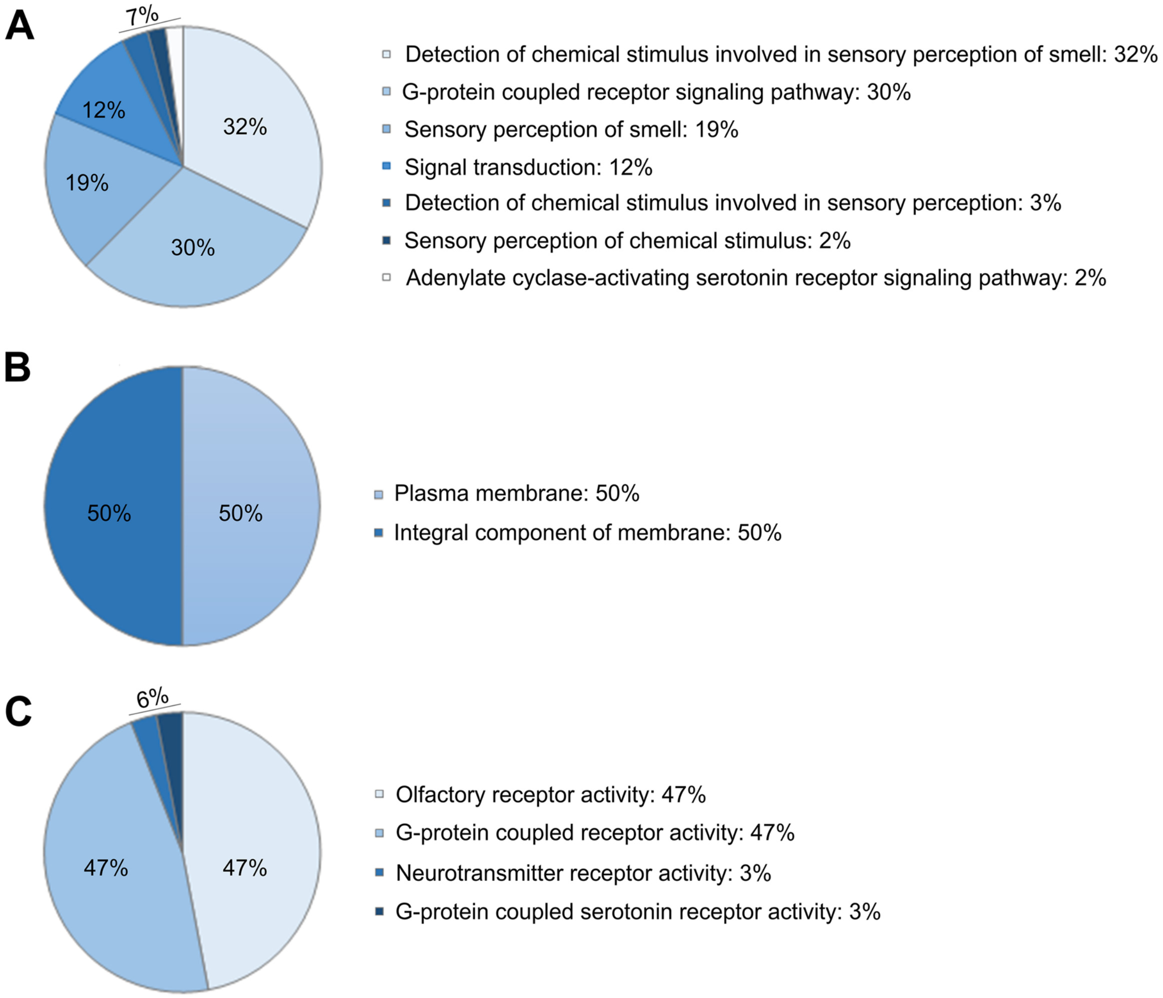

Figure S1. Gene ontology (GO) of common and valid olfactory receptors in cumulus cells. Gene ontology enrichment was performed for comprehensive analysis of selected ORs. Our GO results confirmed OR10H2 function as a serotonin receptor. (A) Top enriched GO terms in the biological process class; (B) Top enriched GO terms in the cellular component class; (C) Top enriched GO terms in the molecular function class, which are selected according to P-value ≤ 0.05.
